# κ-Opioid Receptor Stimulation Improves Endothelial Function in Hypoxic Pulmonary Hypertension

**DOI:** 10.1371/journal.pone.0060850

**Published:** 2013-05-07

**Authors:** Qi Wu, Hai-Yan Wang, Juan Li, Peng Zhou, Qiu-Lin Wang, Lei Zhao, Rong Fan, Yue-Min Wang, Xue-Zeng Xu, Ding-Hua Yi, Shi-Qiang Yu, Jian-Ming Pei

**Affiliations:** 1 Department of Cardiology, Chengdu Medical College, Chengdu, P.R. China; 2 Department of Cardiology, Tangdu Hospital, Fourth Military Medical University, Xi'an, P.R. China; 3 Department of Physiology, National Key Discipline of Cell Biology, Fourth Military Medical University, Xi’an, P.R. China; 4 Department of Cardiosurgery, Xijing Hospital, Fourth Military Medical University, Xi’an, P.R. China; UAE University, Faculty of Medicine & Health Sciences, United Arab Emirates

## Abstract

The present study was designed to investigate the effect of κ-opioid receptor stimulation with U50,488H on endothelial function and underlying mechanism in rats with hypoxic pulmonary hypertension (HPH). Chronic hypoxia-induced HPH was simulated by exposing the rats to 10% oxygen for 2 wk. After hypoxia, mean pulmonary arterial pressure (mPAP), right ventricular pressure (RVP) and right ventricular hypertrophy index (RVHI) were measured. Relaxation of pulmonary artery in response to acetylcholine (ACh) was determined. Expression and activity of endothelial nitric oxide (NO) synthase (eNOS) and inducible NO synthase (iNOS) with NO production, total antioxidant capacity (T-AOC), gp91^phox^ expression and nitrotyrosine content were measured. The effect of U50,488H administration during chronic hypoxia was investigated. Administration of U50,488H significantly decreased mPAP and right ventricular hypertrophy as evidenced by reduction in RVP and RVHI. These effects were mediated by κ-opioid receptor. In the meantime, treatment with U50,488H significantly improved endothelial function as evidenced by enhanced relaxation in response to ACh. Moreover, U50,488H resulted in a significant increase in eNOS phosphorylation, NO content in serum, and T-AOC in pulmonary artery of HPH rats. In addition, the activity of eNOS was enhanced, but the activity of iNOS was attenuated in the pulmonary artery of chronic hypoxic rats treated with U50,488H. On the other hand, U50,488H markedly blunted HPH-induced elevation of gp91^phox^ expression and nitrotyrosine content in pulmonary artery, and these effects were blocked by nor-BNI, a selective κ-opioid receptor antagonist. These data suggest that κ-opioid receptor stimulation with U50,488H improves endothelial function in rats with HPH. The mechanism of action might be attributed to the preservation of eNOS activity, enhancement of eNOS phosphorylation, downregulation of iNOS activity and its antioxidative/nitrative effect.

## Introduction

Pulmonary hypertension (PH) is a chronic disease characterized by progressively increased pulmonary vascular resistance and vascular remodeling and it has been recognized as ‘cancer of cardiovascular diseases’ because of its high mortality and morbidity. So far, there has been no magic cure for pulmonary hypertension, and the goal of treatment is to delay or prevent the progression of this disease. Among various types of PH, hypoxic pulmonary hypertension (HPH) which occurs in patients with cardiopulmonary disease and residents at high altitude has aroused great interest from researchers. Although many investigators have demonstrated that the pathogenesis of the hypoxic pulmonary vasoconstriction has been associated with Kv channel, endothelin-1, serotonin and so on [1]–[3], the precise mechanism remains unclear. In addition, there are limited options available for governing the development of HPH. Therefore, deeply investigating the pathogenesis of HPH and seeking effective control strategy are of great significance.

Hypoxia induced endothelium injury is believed to play a great role in the initiation and development of HPH. When the vascular endothelium was impaired, the balance between a variety of vasomotor factors such as nitric oxide (NO), endothelin (ET) and angiotensin II (AngII) was disturbed, leading to pulmonary vascular vasoconstriction and ultimately pulmonary vascular remodeling in endstage. Impaired production of NO, which exhibits vasorelaxtion effect [4], has long been considered to be a pathogenesis of PH [5], [6]. Reduced NO content can be caused by either blunted NO production or enhanced NO destruction. NO, produced in the endothelial cell, is a product of the enzymatic conversion of L-arginine to L-citrulline by nitric oxide synthase. The conversion functions well in the presence of various cofactors, such as tetrahydrobiopterin, FAD, and FMN [7]. NO can rapidly reacts with superoxide anion (O_2_^−^) to form the toxical oxidant peroxynitrite (ONOO^−^) which can result in oxidative damage, nitration, and S-nitrosylation of biomolecules [8], [9]. Our previous work has demonstrated that U50,488H (a selective κ-opioid receptor agonist) could effectively relax isolated pulmonary artery ring and suppress pulmonary artery pressure of HPH rats. Furthermore, U50,488H has been demonstrated to relax pulmonary artery ring of HPH rats in an endothelium-dependent fashion and this effect can be blunted in the presence of NO synthase inhibitor, which indicate that the effect of U50,488H on relaxing pulmonary is closely related with signaling pathway of NO production. Recent research suggests that NADPH oxidase (major source of superoxide) is a key mediator of HPH and that it contributes to the development of pulmonary vasoconstriction and vascular remodeling [10]. Therefore, strategies aimed at attenuating superoxide may prevent the progression of HPH.

Therefore, the aims of the present study were 1) to investigate whether U50,488H might improve endothelial function of HPH rats and, if so, 2) to investigate the mechanisms involved.

## Materials and Methods

Male Sprague-Dawley rats (200±10 g) from the animal center of the Fourth Military Medical University on Animal Care were used. This study conforms to the Guide for the Care and Use of Laboratory Animals published by the U.S. National Institutes of Health, NIH Publication No. 85–23, revised 1996. Ethical approval for this study was also granted by the University Ethics Committee.

### Animal Models

Rat HPH model was produced as described previously [11], [12]. Briefly, rats were subjected to hypoxia for 8 h every day in a homemade automodulating cabin (air pressure 50 kPa, oxygen concentration 10%). The animals in the control group were kept in room air in the same laboratory. Animals were randomly divided into the following groups: normoxic control group (Con), hypoxia for 2 wk group (2 w), hypoxia for 2 wk+saline group (2 w+NS) (0.3 mL saline was intraperitoneally injected every other day), hypoxia for 2 wk+U50,488H group (2 w+U50) (1.25 mg/kg/day U50,488H was intraperitoneally injected 10 min before hypoxia every other day) [13], hypoxia for 2 wk+U50,488H+nor-BNI group (2 w+U50+nor-BNI) (2.0 mg/kg/day nor-BNI, a selective κ-opioid receptor antagonist, was intraperitoneally injected 20 min before hypoxia and 1.25 mg/kg/day U50,488H was intraperitoneally injected 10 min before hypoxia every other day) and hypoxia for 2 wk+nor-BNI group (2 w+nor-BNI) (2.0 mg/kg/day nor-BNI was intraperitoneally injected 10 min before hypoxia every other day).

### Hemadynamics Measurement and Assessment of Right Ventricular Hypertrophy

Adult male SD rats were anesthetized by intraperitoneal injection of 60 mg/kg pentobarbital sodium. Supplemental doses of sodium pentobarbital were given when needed. The mean pulmonary arterial pressure (mPAP) and right ventricular pressure (RVP) were measured by inserting a polyethylene micro-catheter from right external jugular vein into right ventricle and pulmonary artery as described previously [14]. The mPAP and RVP data were digitally processed via a hemodynamic analyzing system (Chengdu Instrument Co., China). After finishing the experiment, hearts, blood and pulmonary were harvested and then undergone further treatment according to the purpose of the experiment. Then right ventricle (RV), left ventricle (LV), and septum (S) were isolated and RV hypertrophy index (RVHI) was expressed as the tissue weight ratios of RV/(LV+S) and RV/BW (body weight).

### Determination of Endothelial Function

The pulmonary artery was separated gentlely and carefully, and quickly placed in the low temperature Kerb's solution (0∼4°C) in order to maintain its vasoactive activity. After the perivascular fat connective tissues were carefully removed, the pulmonary arteries were cut into 3 mm segments, and quickly placed in the low temperature Kerb's solution (0∼4°C) for further treatment. Pulmonary artery segment was subjected to isolated vascular perfusion system and connected to the tension sensor and the pulmonary artery tension was recorded by multi-channel physiological recording system (RM6280). If the relaxation activity of pulmonary artery in response to ACh (endothelium dependent vasodilators) decreases but has normal relaxation activity in response to NaNO_2_, we may come to a conclusion that endothelial function is impaired. By comparing different responses to two types of vasodilators, we investigate whether U50,488H treatment improves endothelial function.

### Measurements of Nitrate/nitrite Levels in Blood

Blood samples were drawn at the end of 2 wk of hypoxia period. After centrifuging 20 min at 3000 rpm, the supernants were collected. Nitric oxide (NO) can be easily oxidated, leading to the formation of nitrate/nitrite. Thus, nitrate/nitrite levels are accepted as an indicator of NO production. Nitrate/nitrite levels were measured in serum of the rats with a commercially available colorimetric assay kit (nitrate reductase), as previously described [15].

### Immunoblot Analysis

Pulmonary artery tissues were lysed in buffer (1 mM each: antipain, benzamidine, leupeptin, pepstatin A, and phenylmethyl sulfonylfluoride (PMSF), 1% sodium dodecyl sulphate (SDS), and 5 mM ethylene diamine tetraacetic acid (EDTA)). Equal amounts of protein (40 g protein/lane) were electrophoresed on an 8% SDS-polyacrylamide gel and electrophoretically transferred to nitrocellulose membranes (Pall Corporation, Ann Arbor, MI). After blocking with 3% BSA (3% wt/vol) in Tris-buffered saline at room temperature for 1 h, the membranes were incubated with an antibody against eNOS(abcam), iNOS (abcam), or gp91^phox^ (abcam), p-eNOS Ser^1177^, overnight at 4°C. Then, the membranes were washed with PBS and incubated with horseradish peroxidase-conjugated IgG antibody for 1 h at room temperature. The immunoblotting was detected using an enhanced chemiluminescence detection kit (Millipore) with ChemiDocXRS (Bio-Rad Laboratory, Hercules, CA), and the blot densities were analyzed with Quantity One Software.

### Immunohistochemical Localization of 3-Nitrotyrosine (3-NT)

Immunofluorescence detection of 3-nitrotyrosine (Millipore; 1∶200 dilution) in right lung frozen section was conducted as previously described [16]. Samples were treated with H_2_O_2_ (4.5%) to quench/inhibit endogenous peroxidase. After blocking, the sections were reacted with anti-3-NT antibody (Upstate Biotechnology Inc., Lake Placid, NY) for 1 h at room temperature. After extensive washing with PBS, the sections were incubated with antibody peroxidase conjugated for 1 h and finally incubated with diaminobenzidine for 30 min. Quantitative image analysis was performed with image analysis software (Image-Pro Plus 6.0, Media Cybernetics Inc, Bethesda, Maryland, MD, USA). The software determines densitometry mean values of selected tissue regions. Thus, 10 fields/rats were randomly selected, and the intensity of the 3-NT immunostaining was determined.

### eNOS and iNOS Activity Assays

Pulmonary artery tissues were minced and homogenized in 0.9% NaCl solution (1∶10, wt/vol) with a Heidolph DIA900 tissue homogenizer (Heidolph Instruments, Schwabach, Germany). The homogenate was centrifuged (3000 rpm at 4°C for 10 min), the supernatant was decanted, and total NOS activity and inducible NOS (iNOS) activity were determined using an NOS activity assay kit (tNOS, Nanjing Jiancheng Bioengineering Institute) as previously described [17], [18], following the manufacturer’s instructions. eNOS activity was calculated by subtracting iNOS activity from the total NOS activity.

### Tissue Total Antioxidant Capacity (T-AOC) Assay

After rinsed, the pulmonary artery was homogenized in 0.9% NaCl solution (1∶10, wt/vol), and the mixture was subjected to centrifuge at 3000 rpm for 10 min. Then the pellet was discarded and the supernatant was decanted. T-AOC was measured with a spectrophotometric assay kit (Nanjing Jiancheng Bioengineering Institute), according to the manufacture’s instructions.

### Statistical Analysis

Data were presented as mean±SE. The results were analyzed by one way ANOVA followed by Tukey post hoc testing. A value of *P*<0.05 was considered to be statistically significant.

## Results

### U50,488H Attenuated Chronic Hypoxia Induced Elevation in mPAP, RVP and RVHI

**Figure 1:**
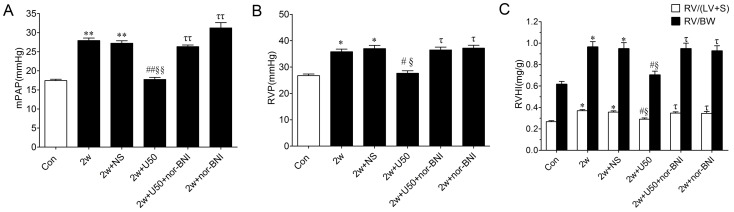
Effect of U50,488H on mPAP, RVP and RVHI in rats exposed to chronic hypoxia for 2 wk. A, group results for mPAP; B, group results for RVP; C, group results for RVHI; Values are means ± SE. n = 8. Con: Control (normoxic group); 2 w: hypoxia for 2 wk; NS: normal saline; U50: U50,488H (1.25 mg/kg); nor-BNI: nor-Binaltorphimine (2.0 mg/kg); mPAP: mean pulmonary artery pressure; RVP: right ventricular pressure; RVHI: right ventricular hypertrophy index. RV: right ventricle; LV: left ventricle; S: septum; BW: body weight. *P<0.05, **P<0.01 vs. control; ^#^ *P*<0.05, ^##^ *P*<0.01 vs. 2 w group; ^§^ *P*<0.05, ^§§^ *P*<0.01 vs. 2 w+NS group; ^τ^ *P*<0.05, ^ττ^ *P*<0.01 vs. 2 w+U50,488H group.

After chronic hypoxia for 2 weeks, mPAP was significantly increased by 57%, and this change was almost abolished by the peritoneal injection of U50,488H, a selective κ-opioid receptor agonist during chronic hypoxia. As shown in Figure1, chronic hypoxia also elicited significantly RV hypertrophy as evidenced by increased RVP, RV/(LV+S) and RV/BW. These changes were significantly attenuated by the peritoneal injection of U50,488H. The effect of U50,488H was abolished by nor-BNI, a selective κ-opioid receptor antagonist, which itself had no effect on these parameters, indicating that U50,488H exerted depressive effect on HPH and RV hypertrophy and this effect was mediated by κ-opioid receptor.

### U50,488H Preserved ACh-induced Vasorelaxation during Chronic Hypoxia

**Figure 2:**
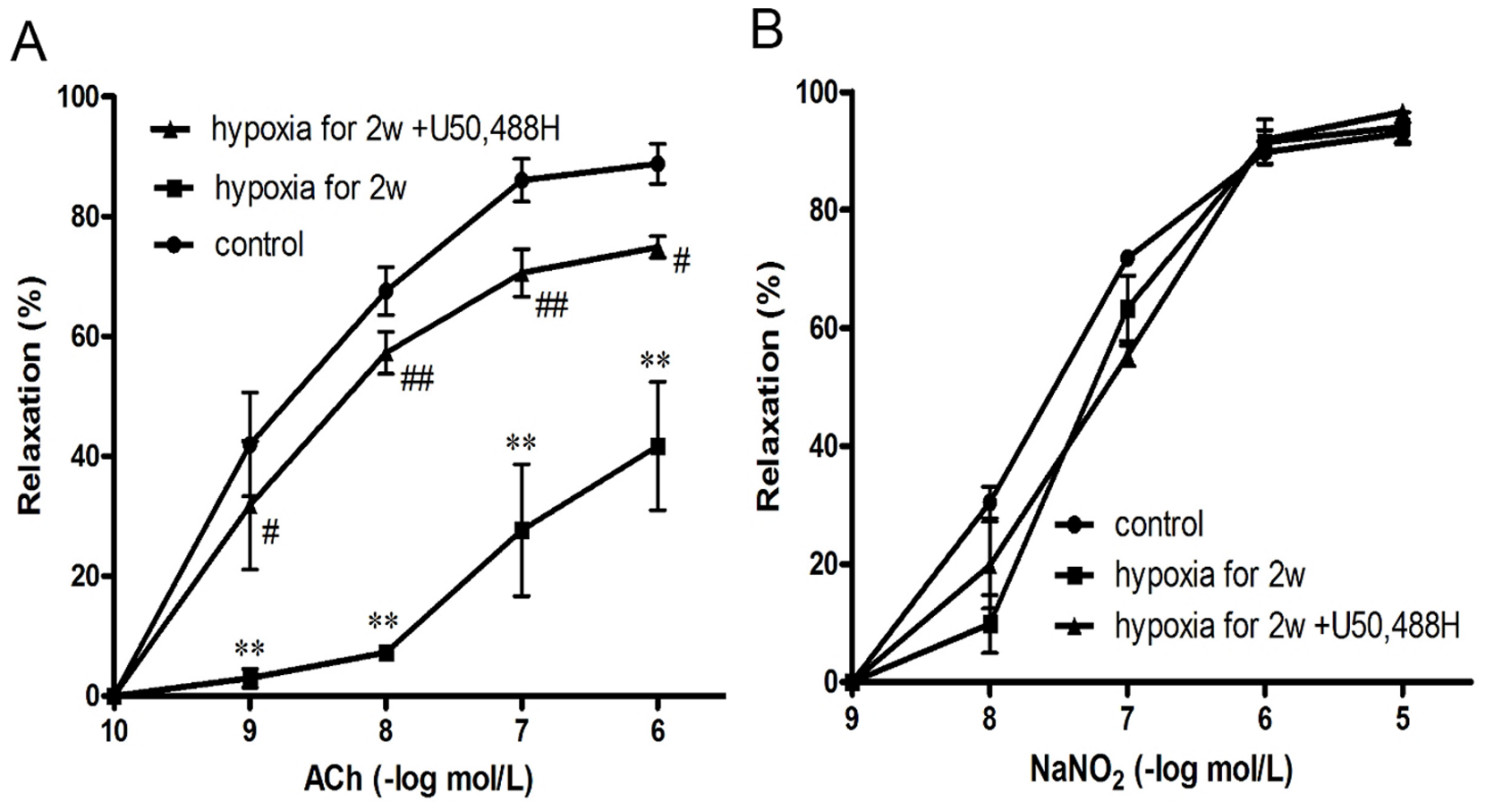
Concentration-dependent vasorelaxation responses to ACh (A) and NaNO_2_ (B) in pulmonary artery segments isolated from rats exposed to normoxia, chronic hypoxia for 2 wk and chronic hypoxia for 2 wk+U50,488H. Con: Control (normoxic group); 2 w: hypoxia for 2 wk; U50: U50,488H (1.25 mg/kg). n = 10 vascular segments/group from 5 rats. **P<0.01 vs. Con; ^#^ *P*<0.05, ^# #^ *P*<0.01 vs. 2 w.

To clarify whether U50,488H could improve endothelial function, isolated pulmonary artery rings (second grade branch of pulmonary artery) in different treatment groups were collected. As shown in Figure 2A, compared with normoxic group, concentration-dependent vasorelaxation in response to ACh was blunted in pulmonary artery isolated from HPH rats (Fig. 2A). However, vasorelaxation in response to the endothelium-independent vasodilator (NaNO_2_) was not altererd (Fig. 2B). These results indicated that chronic hypoxia caused significant endothelial dysfunction. Most intriguingly, treatment with U50,488H for 2 weeks exerted protective roles on endothelial function, as demonstrated by a significant improvement of vasorelaxation in response to ACh.

### U50,488H Reversed Chronic Hypoxia Induced Production of NO in HPH Rats

To investigate the precise mechanism of the improvement of endothelial function, NO content was measured. As demonstrated in Table 1, chronic hypoxia for 2 weeks significantly attenuated serum NO content. More importantly, administration of U50,488H significantly elevated the levels of NO, which was blocked by nor-BNI, indicating that U50,488H stimulated NO production and this effect was mediated by κ-opioid receptor.

**Table 1:** Effect of U50,488H on NO production in serum of HPH rats exposed to chronic hypoxia for 2 wk.

Group	NO(µmol/L)
Control	58.3±2.8
2 w	32.7±2.5 **
2 w+NS	35.2±2.2**
2 w+U50	54.1±2.8^ ##^ §§
2 w+U50+nor-BNI	36.0±2.9ττ
2 w +nor-BNI	36.9±3.4ττ

Values are means ± SE; n = 8. NOx concentration in serum was determined by nitrate enzyme reverting method. 2 w: hypoxia for 2 wk; NS: normal saline; U50: U50,488H; nor-BNI: nor-Binaltorphimine;

** *P*<0.05 vs. control;

## *P*<0.05 vs. 2 w;

§§ *P*<0.05 vs. 2 w+NS;

ττ *P*<0.05 vs. 2 w+U50.

### U50,488H did not Alter eNOS or iNOS Expression in Pulmonary Arteries of HPH Rats

**Figure 3:**
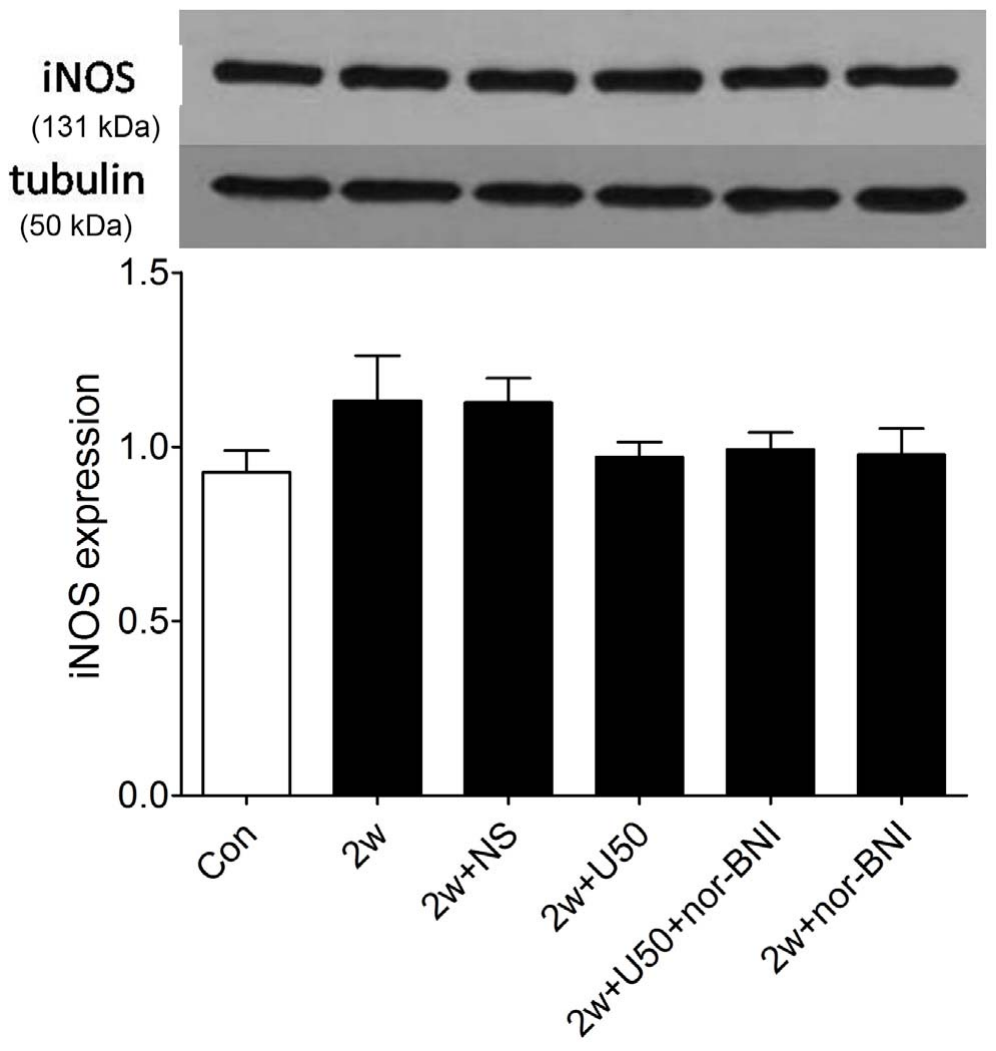
Effect of U50,488H on iNOS expression in pulmonary arteries of HPH rats. Con: control; 2 w: 2 wk hypoxia; 2 w+NS: 2 w+normal saline; 2 w+U50: 2 w+U50,488H; 2 w+U50+nor-BNI: 2 w+U50,488H+nor-BNI; 2 w+nor: 2 w+nor-BNI. All values were expressed as means ± SE, n  = 5 in each group.

We next investigated whether U50,488H-induced increase of NO generation was caused by enhanced eNOS or iNOS protein expression level. As demonstrated in Figure 3, there was little effect on the expression of iNOS in the pulmonary arteries of chronic hypoxic rats after the injection of U50,488H, suggesting that the increased NO production was not derived from the increase in iNOS expression.

**Figure 4:**
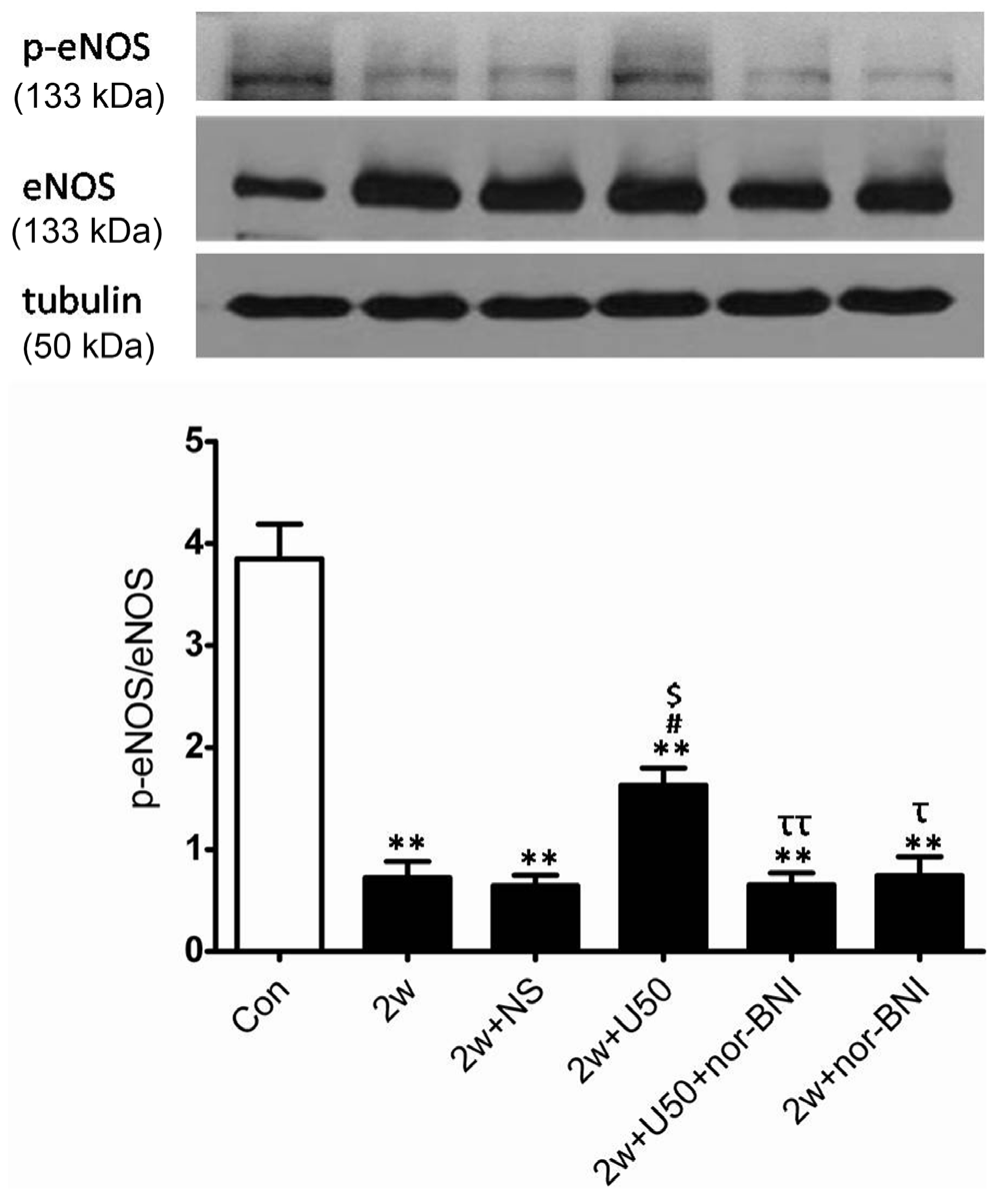
Effect of U50,488H on eNOS expression and phosphorylation in pulmonary arteries of HPH rats. Con: control; 2 w: 2 wk hypoxia; 2 w+NS: 2 w+normal saline; 2 w+U50: 2 w+U50,488H; 2 w+U50+nor-BNI: 2 w+U50,488H+nor-BNI; 2 w+nor: 2 w+nor-BNI. All values were expressed as means ± SE. n = 5. **P<0.01 vs. Con; ^#^ *P*<0.05 vs. 2 w; ^$^ *P*<0.05 vs.2 w+NS; ^ττ^ *P*<0.01 vs. 2 w+U50.

As shown in Figure 4, chronic hypoxia significantly elevated the expression of eNOS, which was not further altered by U50,488H (Fig. 4). Collectively, these results indicated that the increased NO production with U50,488H administration was not attributed to the alteration of eNOS or iNOS expression and there might be other explanations that could account for the discrepancies between impaired NO production and elevated eNOS expression in chronic hypoxia.

### U50,488H Enhanced eNOS Phosphorylation

The mechanism of U50,488H-induced elevation of NO production in HPH rats remains unclear, we, therefore, sought to investigate whether U50,488H exerted this effect via activating eNOS/NO pathway. As shown in Figure 4, chronic hypoxia significantly impaired eNOS phosphorylation, administration of U50,488H significantly increased eNOS phosphorylation and this effect was blocked by nor-BNI. These data indicated that U50,488H increased the phosphorylation level of eNOS.

### U50,488H Enhanced eNOS Activity but Attenuated iNOS Activity

**Figure 5:**
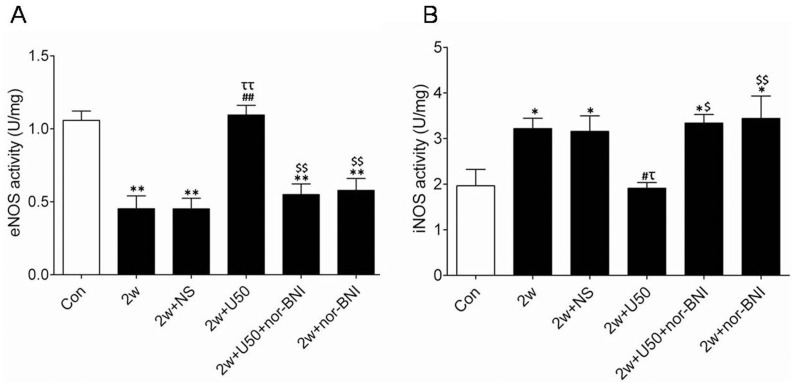
Effect of U50,488H on eNOS and iNOS activity in pulmonary arteries of HPH rats. A, group results for eNOS activity; B, group results for iNOS activity; Con:control; 2 w: 2 wk hypoxia; 2 w+NS: 2 w+normal saline; 2 w+U50: 2 w+U50,488H; 2 w+U50+nor-BNI: 2 w+U50,488H+nor-BNI; 2 w+nor: 2 w+nor-BNI. All values were expressed as means ± SE, n  = 7 in each group. *P<0.05, **P<0.01vs. Con; ^#^ *P*<0.05, ^##^ *P*<0.01 vs. 2 w; ^τ^ *P*<0.05, ^ττ^ *P*<0.01 vs. 2 w+NS; ^$^ *P*<0.05, ^$$^ *P*<0.01 vs. 2 w+U50.

In order to elucidate the precise mechanism of the stimulative role of U50,488H on NO release, eNOS and iNOS activity were measured respectively. As demonstrated in Figure 5, chronic hypoxia significantly decreased eNOS activity and elevated iNOS activity, and administration of U50,488H significantly elevated eNOS activity and decreased iNOS activity. These results indicated that U50,488H might stimulate bioactive NO production by dural regulation of eNOS activity and iNOS activity.

### U50,488H Blunted Hypoxia Induced Enhancement of Superoxide Production

**Figure 6:**
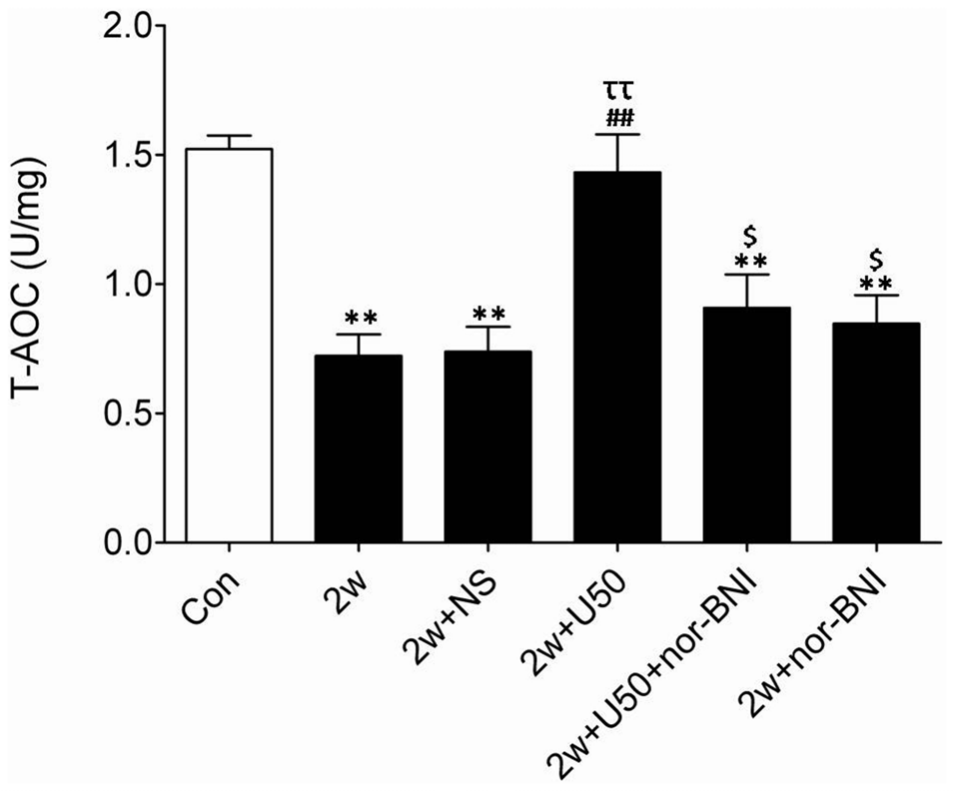
Effect of U50,488H on T-AOC in pulmonary arteries of HPH rats. Con: control; 2 w: 2 wk hypoxia; 2 w+NS: 2 w+normal saline; 2 w+U50: 2 w+U50,488H; 2 w+U50+nor-BNI: 2 w+U50,488H+nor-BNI; 2 w+nor: 2 w+nor-BNI. All values were expressed as means ± SEM, n  = 7 in each group. **P<0.01 vs. Con; ^##^ *P*<0.01 vs. 2 w; ^ττ^ *P*<0.01 vs. 2 w+NS; ^$^ *P*<0.05 vs. 2 w+U50.

**Figure 7:**
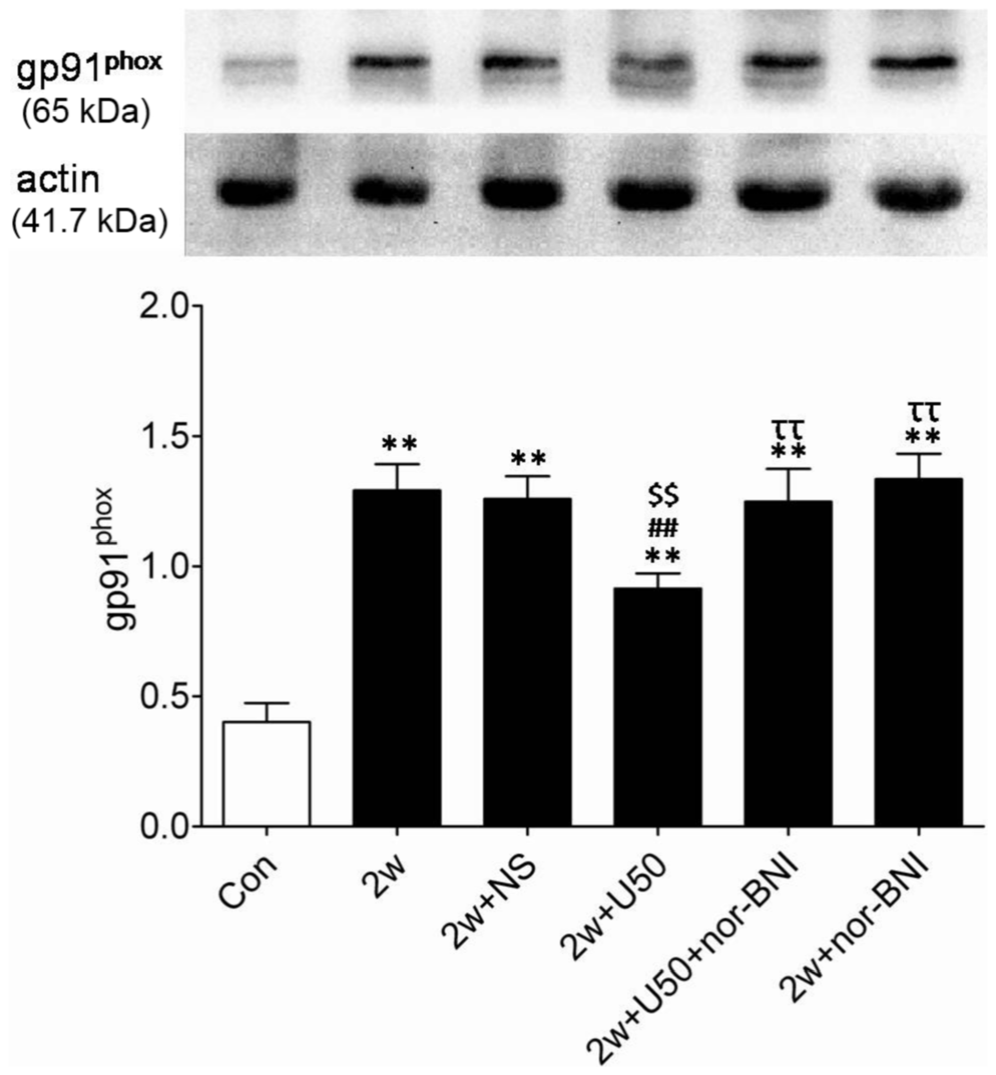
Effect of U50,488H on gp91^phox^ in pulmonary arteries of HPH rats. Con:control; 2 w: 2 w hypoxia; 2 w+NS: 2 w hypoxia+normal saline; 2 w+U50: 2 w hypoxia+U50,488H; 2 w+U50+nor-BNI: 2 w hypoxia+U50,488H+nor-BNI; 2 w+nor: 2 w hypoxia+nor-BNI. All values were expressed as means ± SEM, n  = 4 in each group. **P<0.01 vs. Con; ^##^ *P*<0.01 vs. 2 w; ^$$^ *P*<0.05 vs. 2 w+NS; ^ττ^ *P*<0.01 vs. 2 w+U50.

The data presented above indicate that U50,488H can stimulate bioactive NO production by regulation of eNOS and iNOS activity. However, it remains unclear whether U50,488H can prevent NO destruction. As we all know, the decrease of total antioxidant capacity (T-AOC) may lead to NO destruction. As summarized in Figure 6, chronic hypoxia led to a significant reduction of T-AOC in pulmonary arteries, indicating that chronic hypoxia triggered NO destruction. More importantly, administration of U50,488H significantly elevated T-AOC of pulmonary arteries, indicating that U50,488H may prevent NO destruction. To further support this notion, additional experiments were performed. gp91^phox^, a major source of superoxide, was detected by Western blotting. As showed in Figure 7, consistent with previously published results [19], gp91^phox^ expression was significantly increased in pulmonary arteries of HPH rats. Administration of U50,488H caused a moderate, yet statistically significant, reduction in gp91^phox^ expression. Moreover, this effect was abolished by nor-BNI, indicating that the reducing effect of U50,488H on gp91^phox^ expression was mediated by κ-opioid receptor.

### U50,488H Attenuated ONOO^−^ Production in Pulmonary Artery

**Figure 8:**
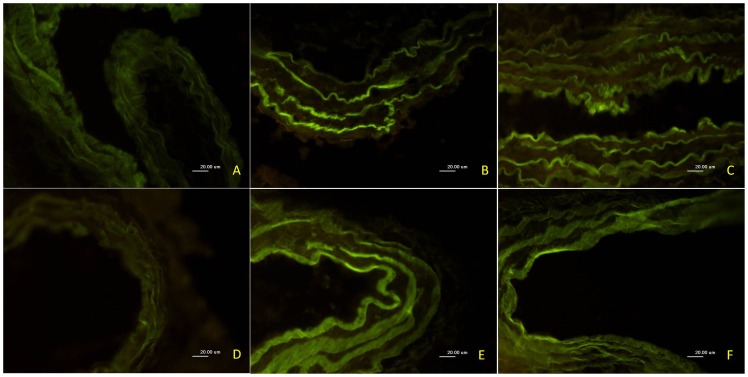
Effect of U50,488H on 3-nitrotyrosine expression in pulmonary arteries of HPH rats. Inserted are representative photographs of 3-nitrotyrosine expression in pulmonary arteries of HPH rats by immunofluorescence (400×). A, control; B, 2 w hypoxia; C, 2 w+normal saline; D, 2 w+U50,488H; E, 2 w+U50,488H+nor-BNI; F, 2 w+nor-BNI.

To obtain more evidence to support this notion that U50,488H prevent NO destruction, ONOO^−^, an index of nitrative stress, which was the biradical reaction product of superoxide and NO, was determined by measurement of nitrotyrosine. As shown in Figure 8, nitrotyrosine immunofluorescence intensity was significantly increased in pulmonary arteries of HPH rats. Administration of U50,488H significantly attenuated nitrotyrosine production. Moreover, this effect was significantly abolished by nor-BNI, indicating that U50,488H exerted anti-nitrative stress effect and this effect was mediated by κ-opioid receptor.

## Discussion

In the present study, chronic treatment with U50,488H was demonstrated to ameliorate endothelial dysfunction in chronic hypoxia. This conclusion is based on the following observations:1) relaxation response of pulmonary artery to ACh but not to NaNO_2_ was markedly ameliorated by U50,488H, 2) U50,488H stimulated bioactive NO production via preservation of eNOS activity and enhancement of eNOS phosphorylation and 3) U50,488H prevented NO destruction by elevating total antioxidant capacity, decreasing gp91^phox^ and ONOO^−^ formation of pulmonary artery.

Our study provides the first evidence that U50,488H, a κ-opioid receptor agonist, significantly attenuated chronic hypoxia-induced endothelial dysfunction. This result holds potential promises that therapeutic application of U50,488H may represent a useful treatment of cardiovascular diseases with vascular disorders. Consistent with previous studies [11], the present study demonstrates that κ-opioid receptor agonist U50,488H significantly reduced mPAP, RVP and RVHI, exerting its protective roles on pulmonary circulation. In this experiment, we, for the first time, observed that the effect of U50,488H-induced attenuation of mPAP, RVP and RVHI was blocked by nor-BNI, a selective κ-opioid receptor antagonist, indicating that this effect was mediated by κ-opioid receptor, and this testified that κ-opioid receptors may be involved in the regulation of pulmonary circulation [12].

HPH is a severe clinical disease, which is characterized by hypoxic pulmonary vasoconstriction and pulmonary vascular remodeling [20]. When the body is exposed to hypoxic environment, vascular endothelium is impaired, leading to the unbalance of the release of various endothelial vasoactive substances. Among these vasoactive substances, NO is of great importance in the etiology of HPH. Attenuated production of NO has been recognized as an important pathogenic factor of HPH [5], [6]. Consistent with previous study, the present study has demonstrated that the level of NO in HPH rats was significantly reduced, and in the meantime, isolated vascular perfusion result demonstrated that chronic hypoxia caused pulmonary endothelial dysfunction. We believe that hypoxia-induced endothelial dysfunction and hypoxia-induced impaired production of NO may share reciprocal causation relationship. In the present study, U50,488H is demonstrated to reverse NO reduction caused by hypoxia and to ameliorate endothelial dysfunction induced by hypoxia. Although there was no direct or solid evidence that U50,488H ameliorated hypoxia-induced endothelial dysfunction by stimulating NO production, we have no reason to deny its close relationship. Additionally, our previous study has demonstrated that the relaxing effect of U50,488H on pulmonary artery was partially blunted by L-NAME, an eNOS inhibitor [21], indicating that the relaxing effect may be related to NO. Based on these findings, we believe that U50,488H-induced elevation of NO may contribute to its role in the improvement of endothelial dysfunction.

NO is generated from L-arginine through enzymatic conversion by NOS synthase [22]–[25]. eNOS/NO pathway involves a variety of mechanisms: 1. protein-protein interactions, for example, enhanced interaction between eNOS and calmodulin/HSP90 up-regulates eNOS activity, however, enhanced interaction between eNOS and caveolin-1 reduces eNOS activity [7], [26], [27]; 2. modification of protein such as phosphorylation, for example, protein kinase A and Akt can lead to eNOS phosphorylation and thus elevation of eNOS activity [28]–[30], however, PKC induced eNOS phosphorylation downregulates eNOS activity [31]; eNOS expression has been reported to increase [32], [33] or decrease [34], [35] upon hypoxia stimuli by different research groups. In the present study, we found that chronic hypoxia for 2 wk upregulated eNOS protein expression, but its phosphorylation levels were significantly reduced. Discrepancies of eNOS expression in PH may be due to the stage of the disease, different model and different cell types.

Xu et al has demonstrated that κ-opioid receptor and caveolin-1 are coexpressed in lipid rafts [36], indicating that there may be possible interactions between them. Besides, Patel et al have identified the co-localization of the δ-opioid receptors and caveolin-3 and have demonstrated that activation of δ-opioid receptor may confer myocardial protection via a caveolin-dependent fashion [37]. Most importantly, Murata et al have demonstrated that reduction of eNOS activity in HPH may result from enhanced interaction between eNOS and caveolin-1 [38]. Our experiments also demonstrated that eNOS activity was reduced in HPH rats and U50,488H increased eNOS activity. However, it still remains unclear whether activation of the κ-opioid receptor with U50,488H can affect the interaction between eNOS and caveolin-1. In addition, it still warrants further investigation whether there are interactions between caveolin-1 and κ-opioid receptor. In this study, eNOS activity can be restored nearly 100% by U50,488H whereas eNOS phosphorylation barely reached about 50% recovery. It has been reported that eNOS can be activated by stimuli as a consequence of an increase in the [Ca^2+^]_i_ and altered eNOS phosphorylation [39], indicating that eNOS phosphorylation is not a single factor accounting for the alteration of eNOS activity. Since it has been well demonstrated that U50,488H may affect [Ca^2+^]_i_, the latter may also account for the changes of eNOS activity. Further study is needed to confirm this speculation.

In this study, we found that neither HPH nor U50,488H altered iNOS expression but they significantly changed iNOS activity. Study by Sharon et al [40] showed that hypoxia inhibits NO production without altering iNOS protein expression. Now we have also found that hypoxia and U50,488H regulated iNOS activity without changing its expression. This phenomenon proved that changes of iNOS activity sometimes might not be necessarily accompanied by changes of iNOS expression The underlying mechanism needs further study.

Accumulating evidence now suggests that NADPH oxidase is the major source of superoxide in vascular tissues [41]. Nisbet et al have confirmed that NADPH oxidase-derived ROS participates in chronic hypoxic pulmonary vascular remodeling and HPH [10]. In hypoxia, superoxide (O_2_^−^) reacts with NO to form peroxynitrite (ONOO^−^) and other reactive nitrogen species, effectively reducing NO bioavailability. ONOO^−^ is a highly reactive species and can initiate both nitrosative and oxidative reactions in vitro and in vivo. A characteristic reaction of ONOO^−^ is the nitration of protein-bound tyrosine residues to generate 3-nitrotyrosine-positive proteins [42], [43]. In this study, result showed that increased 3-nitrotyrosine-positive proteins in pulmonary artery of HPH rats was significantly attenuated after U50,488H treatment. So we conclude that U50,488H may play a role in both antinitrosative and antioxidative reactions in HPH rats. Additionally, oxidative stress and nitrative stress are involved in the pathogenesis of HPH. More importantly, studies have demonstrated that superoxide dismutase and NADPH oxidases inhibitors can significantly blunt acute hypoxia induced pulmonary vasoconstriction [44], [45]. Based on the studies mentioned above, we believe that the down-regulation of ROS may be a treatment strategy against HPH. In our present study, direct evidence was obtained to indicate that U50,488H improves endothelial function by its antioxidative/antinitrative property. First, chronic hypoxia-induced reduction of total antioxidant capacity (T-AOC) was significantly reversed by treatment with U50,488H. It is known to all that superoxide reacts rapidly with NO, leading to the destruction of NO and the formation of toxic ONOO^−^ [42]. This evidence implies that increased ONOO^−^ formation in pulmonary artery of HPH rats was significantly attenuated after U50,488H treatment. Second, the present study has demonstrated that chronic treatment with U50,488H significantly reduced gp91^phox^ (an important component of NADPH oxidase) expression in pulmonary artery of HPH rats. Collectively, our study has obtained evidence that U50,488H ameliorates endothelial dysfunction induced by chronic hypoxia, at least in part by its antioxidative and antinitrative property that have never been previously identified.

In summary, we have demonstrated for the first time that U50,488H may confer endothelium protection, at least partly via its promotion on NO production and its antioxidant/nitrative properties which prevent NO destruction. The results not only provide new ideas regarding the relationship between opioid receptor and HPH, but also highlight U50,488H as a potential agent against HPH in clinical practice.
